# Point-of-Care Ultrasound Integrated With Teleconsultation for Rural Home-Based Medical Care: Pilot Implementation and Financial Analysis

**DOI:** 10.2196/93876

**Published:** 2026-08-28

**Authors:** Hung-Bin Tsai, Nin-Chieh Hsu, Sang Ju Yu, Feng-Jung Yang

**Affiliations:** 1 Department of Internal Medicine National Taiwan University Hospital Taipei, Taiwan Taiwan; 2 College of Medicine National Taiwan University Taipei, Taiwan Taiwan; 3 Home Clinic Dulan Taitung Taiwan; 4 Department of Medical Genetics and Department of Internal Medicine National Taiwan University Hospital Taipei, Zhongzheng District Taiwan

**Keywords:** point-of-care ultrasound, teleconsultation, tele-ultrasound, home health care, hospital at home, rural health services, telemedicine, financial analysis, aged, Taiwan

## Abstract

**Background:**

Population aging and geographic health disparities challenge health care delivery in rural settings worldwide. Point-of-care ultrasound (PoCUS) combined with teleconsultation may enhance home-based medical care by extending specialist expertise to underserved communities, yet evidence from real-world implementation in Asian settings remains scarce. Taiwan, with its high digital literacy and universal National Health Insurance (NHI) system, provides a unique context for evaluating integrated PoCUS-teleconsultation service delivery.

**Objective:**

This study aimed to describe the implementation, clinical applications, and financial sustainability of an integrated PoCUS-teleconsultation service within a dedicated home-based medical care practice in rural eastern Taiwan.

**Methods:**

We conducted a retrospective analysis of prospectively recorded routine care data at D Clinic, Taitung County, from April 2020 to May 2022. PoCUS examinations conducted in the clinic, during home visits, and through mobile outreach were recorded. A business-to-business-to-consumer (B2B2C) teleconsultation model linking on-site physicians with remote specialists via real-time ultrasound streaming was implemented from March 2021. Seven-year net present value (NPV) analyses evaluated financial viability across 24 equipment reimbursement scenarios.

**Results:**

Among the 26 patients who received teleconsultation, the mean age was 79.4 (SD 9.2) years, 15 (58%) were male, and 20 (77%) were in the intensive home health care tier. A total of 676 PoCUS examinations (defined as single-region imaging studies) were performed by 3 trained physicians in the following settings: 375 (55.5%) in the clinic, 174 (25.7%) during home visits, and 127 (18.8%) as mobile outreach. Twenty-six teleconsultation sessions were conducted (n=15, 58% during home visits; n=7, 27% during mobile outreach; and n=4, 15% in the clinic), with 13 (50%) involving multitarget scanning (cardiac, pulmonary, and abdominal). Of the 26 sessions, 24 (92%) were completed without technical interruption, while 2 (8%) experienced transient 4G buffering but were completed. Monthly PoCUS volume reached a sustained plateau of ≥28 examinations from month 17 of implementation. Four clinical service domains were identified: hospital-at-home acute management, home-based hospice multiorgan support, green channel surgical referral, and chronic disease serial monitoring. NPV analysis demonstrated that wireless PoCUS equipment with the proposed, but not yet implemented, dual-specialist teleconsultation reimbursement achieved the most favorable projected return (NT $882,000; NT $1=US $0.036 in 2021 on average), requiring only 2.72 monthly cases to break even under the modeled assumptions.

**Conclusions:**

Integrating PoCUS with teleconsultation is operationally feasible in rural home-based care when wireless equipment is adopted. Financial scenario modeling suggests favorable projected returns under proposed reimbursement structures, although these remain contingent on NHI policy adoption. These findings are hypothesis generating and require validation through multisite studies with patient-level outcome measurement before informing national reimbursement policy.

## Introduction

The global demographic transition toward aging populations presents unprecedented challenges for health care systems, particularly in countries experiencing rapidly declining fertility rates and growing populations of older adults [1]. Taiwan exemplifies this demographic urgency: the National Development Council projected that by 2025, 1 in 5 citizens would be aged ≥65 years, classifying Taiwan as a “superaged” society—the fastest demographic transition among high-income nations [2]. In rural and mountainous regions such as Taitung County in eastern Taiwan, these challenges are compounded by physician shortages, limited specialist access, and transportation barriers that disproportionately affect homebound older adult populations.

The hospital-at-home (HaH) model has emerged as a promising strategy to address these disparities by delivering acute and chronic care services in patients’ residences [3,4]. Meta-analyses have demonstrated that HaH programs reduce mortality, readmission rates, and health care costs while improving patient and caregiver satisfaction [3]. Recent evidence from Israel’s largest health maintenance organization demonstrated that integrating telemedicine into HaH services enhanced clinical decision-making and reduced unnecessary emergency department use [5]. However, the diagnostic capabilities of HaH programs remain limited by the absence of bedside imaging—a gap that point-of-care ultrasound (PoCUS) is uniquely positioned to fill.

PoCUS has evolved from a hospital-centric diagnostic modality to a portable, clinician-performed bedside tool with broad applications across emergency, critical care, and primary care settings [6-8]. Position statements from the Society of Hospital Medicine and the Society of General Internal Medicine now endorse PoCUS as a core competency for hospitalists, encompassing cardiac, pulmonary, abdominal, vascular, and musculoskeletal domains [9-11]. The indication, acquisition, interpretation, and medical decision-making (I-AIM) framework provides a structured approach to PoCUS documentation and quality assurance [12,13].

Tele-ultrasound—the remote guidance and interpretation of ultrasound examinations—has shown promise in extending specialist expertise to underserved settings. A systematic review by Kariman et al [14] of 31 studies in pregnancy care found fetal tele-ultrasound services (11 studies) to be feasible and particularly valuable in rural areas and in settings where specialist care is centralized, although study quality was generally low to fair and the evidence was confined to obstetric applications. A comparable synthesis of home-based, multiorgan tele-ultrasound in adults is lacking. Kirkpatrick et al [15] demonstrated that novice operators performing PoCUS in home environments during the COVID-19 pandemic could achieve diagnostic-quality images with remote expert guidance, suggesting that tele-mentored PoCUS is feasible even with limited prior training.

Taiwan’s telemedicine regulatory framework has evolved substantially since its initial legislation in 1986, with landmark amendments in 2018 permitting teleconsultation for patients in remote areas, long-term care facilities, and emergency situations, and further expansion in 2024 removing geographic restrictions entirely [16,17]. The COVID-19 pandemic catalyzed a 72-fold surge in National Health Insurance (NHI) teleconsultation claims, from 2330 cases in 2019 to approximately 169,044 in 2022 [18,19]. However, the existing NHI reimbursement structure lacks dedicated codes for multitarget bedside ultrasound in home settings and does not recognize business-to-business (B2B) specialist teleconsultation fees—creating a policy gap that threatens the financial sustainability of integrated PoCUS-teleconsultation services.

Notably, South Korea provides a regional precedent: following the introduction of dedicated PoCUS reimbursement codes (single-target ultrasound [STU] and multitarget ultrasound [MTU]) in July 2019, Kang et al [20] documented a 4-fold increase in emergency department PoCUS use, demonstrating that reimbursement policy directly drives adoption [21].

Taiwan’s high smartphone penetration rate (exceeding 85% among adults) and widespread 4G or 5G network coverage, including in rural eastern regions, provide a favorable digital infrastructure foundation for teleconsultation implementation. The business-to-business-to-consumer (B2B2C) model extends conventional physician-to-physician teleconsultation by adding a direct patient-facing service delivery component, operationally distinguishing it from standard telemedicine by embedding the remote consultation within a structured home visit workflow rather than replacing the in-person encounter.

Despite growing interest in both PoCUS and telemedicine, to our knowledge, no published study has systematically examined the integration of PoCUS with specialist teleconsultation within a dedicated home-based medical care practice, nor has any study provided financial modeling to assess the sustainability of such integration under different reimbursement scenarios. This study aimed to fill these gaps by reporting the implementation experience, clinical applications, learning curve dynamics, and economic analysis of a PoCUS-teleconsultation service at D Clinic, a dedicated home-based medical care practice in Taitung County, eastern Taiwan.

## Methods

### Study Design and Setting

We conducted a retrospective analysis of prospectively recorded routine care data at D Clinic, the first dedicated home-based medical care clinic in Taitung County, eastern Taiwan, established in December 2017. The study period for PoCUS use spanned April 2020 to May 2022, with teleconsultation services initiated in March 2021 during the Delta and Omicron waves of the COVID-19 pandemic. The clinic serves approximately 90 home health care patients and manages 120 long-term care disability assessment cases across Dulan, Xingchang, and Longchang villages and is staffed by 3 physicians (2 in family medicine and 1 in neurology) and 1 nursing case manager.

### Patient Population and Care Tiers

Patients were enrolled in 3 NHI-defined care tiers: (1) home health care (Barthel Index <60), (2) intensive home health care (bedridden for ≥50% of waking hours and requiring skilled nursing), and (3) home-based hospice (terminal conditions meeting NHI criteria). Clinical indications for PoCUS-teleconsultation included acute decompensation of chronic conditions, suspected surgical emergencies, end-of-life symptom management requiring multiorgan assessment, and chronic disease monitoring requiring specialist input for medication adjustment.

### PoCUS Equipment and Teleconsultation Platform

Both wired and wireless ultrasound systems were evaluated during the study period (Table 1). Equipment selection criteria included portability (<400 g for wireless devices), battery life, ease of disinfection, imaging modes (B-mode, M-mode, and color Doppler), and connectivity for real-time image streaming. Teleconsultation was conducted using a hybrid platform combining LINE (LY Corp) video calling and the National Health Research Institutes dedicated teleconsultation platform via 4G cellular networks. Remote specialists included a cardiologist (at Kaohsiung Chang Gung Memorial Hospital) and a hospitalist (in integrative medicine at National Taiwan University Hospital). The I-AIM framework was adopted for standardized PoCUS documentation and quality assurance [12,13].

**Table 1:** Point-of-care ultrasound equipment specifications and cost comparison.

Parameters	Option A: wired phased-array+linear	Option B: wired convex+linear	Option C: wireless phased-array+linear	Option D: wireless convex+linear
Representative device	BENQ T3300	BENQ T3300	Philips Lumify or GE Vscan air	Philips Lumify or GE Vscan air
Weight	2.7 kg (system)	2.7 kg (system)	<400 g (probe only)	<400 g (probe only)
Probe types	Phased-array+linear	Convex+linear	Phased-array+linear	Convex+linear
Imaging modes	B-mode, M-mode, and color Doppler	B-mode, M-mode, and color Doppler	B-mode, M-mode, and color Doppler	B-mode, M-mode, and color Doppler
Cost (NTD)^a^	1 million	1 million	550,000	500,000
Probe depreciation (years)	7	7	7	7
Portability rating	Moderate	Moderate	Excellent	Excellent
Disinfection ease	Moderate	Moderate	High	High

^a^NT $1=US $0.036 in 2021 on average. Costs reflect 2021 procurement prices. Depreciation follows the standard National Health Insurance medical equipment schedule.

### B2B2C Teleconsultation Model

**Figure 1:**
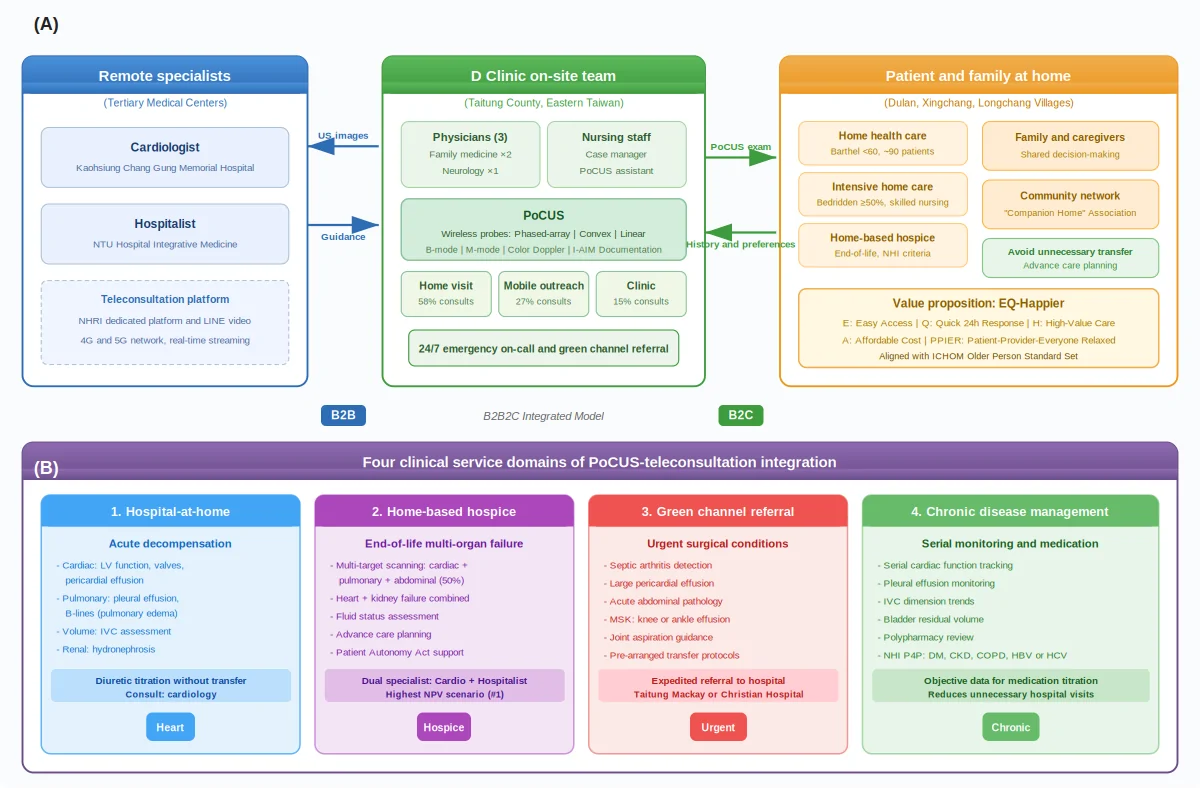
Business-to-business-to-consumer (B2B2C)–integrated point-of-care ultrasound (PoCUS)–teleconsultation model for home-based medical care. (A) Three-tier model showing remote specialists (business-to-business [B2B]), the D clinic on-site team (hub), and patients and families at home (business-to-consumer [B2C]). (B) Four clinical service domains identified through case-review analysis. I-AIM: indication, acquisition, interpretation, and medical decision-making; ICHOM: International Consortium for Health Outcomes Measurement; NHI: National Health Insurance.

The teleconsultation workflow followed a B2B2C model (Figure 1A): the on-site D Clinic physician performed PoCUS examinations at the patient’s home, streamed real-time ultrasound images and clinical findings to remote specialists (B2B component), who provided diagnostic interpretation and therapeutic recommendations that were communicated back to the patient and family (business-to-consumer [B2C] component). Sessions were categorized by consultation type: image interpretation with medication adjustment and image interpretation only. Dual-specialist consultations (cardiologist and hospitalist) were arranged for complex end-of-life cases involving multiorgan failure.

### Data Collection and Outcomes

Data were recorded routinely during clinical care and retrospectively compiled for this analysis; they included (1) monthly PoCUS examination counts by clinical setting (clinic, home visit, and mobile outreach) and examination type (abdominal, bladder, cardiac, musculoskeletal, thyroid, and extremity); (2) teleconsultation session characteristics, including specialist type, consultation duration, multitarget scanning status, and clinical indication; (3) qualitative learning curve indicators, including temporal trends in monthly volume, scope expansion, and communication competency milestones; and (4) clinical service domain categorization based on case-review analysis. Each PoCUS examination was defined as a single imaging study targeting 1 anatomical region (eg, cardiac, pulmonary, abdominal, or vascular). A single patient encounter could generate multiple examinations if multiple regions were scanned; the 676 examinations therefore represent imaging studies, not unique patient encounters. Billing classification followed NHI reimbursement rules: examinations qualifying under existing NHI ultrasound codes—abdominal ultrasound (19001C, 882 points), follow-up ultrasound (19009C, 643 points), and head-and-neck ultrasound for thyroid studies (19012C, 610 points)—were classified as billed, whereas point-of-care examinations reported under the generic “other ultrasound” code (19005C, 600 points) and abdominal screening performed without a specific coded indication were classified as unbilled. The clinic did not bill echocardiography (18005C). Clinical service domains were identified through retrospective case-review analysis conducted independently by both authors using a consensus-based approach. Teleconsultation sessions were initiated at the discretion of the on-site physician when clinical findings exceeded the scope of independent management or when multiorgan assessment required specialist input; no prespecified severity threshold was applied. All 26 sessions during the study period are reported; no eligible cases were excluded. The teleconsultation trigger rate was defined as the proportion of PoCUS examinations that prompted a real-time remote specialist consultation.

### Financial Analysis

A 7-year net present value (NPV) analysis was performed to evaluate financial viability across 24 scenarios combining 4 equipment options (Table 1) with 6 consultation reimbursement structures, using the cost and volume parameters of the source financial model developed for this service [22]. Equipment costs (NT $1=US $0.036 in 2021 on average) ranged from NT $500,000 (wireless convex and linear) to NT $1 million (wired phased-array and linear or wired convex and linear), with the ultrasound probe depreciated over 7 years and ancillary electronic equipment over 3 years (Table S5 in Multimedia Appendix 1). All 24 scenarios, together with the record of their verification against that model, are provided in Multimedia Appendix 2. Proposed reimbursement rates included NT $3000 per remote specialist session (image interpretation and medication adjustment) or NT $2000 (image interpretation only), plus NT $1200 for an on-site multitarget PoCUS examination. Case volume projections followed: year 1, 26 cases; year 2, 57; year 3, 80; and steady-state (years 4-7), 100 cases annually. A 6% discount rate was applied. Break-even analysis identified the minimum monthly case volume required for each scenario. Under the current NHI framework, no dedicated B2B teleconsultation fee exists; the proposed reimbursement rates used in modeling reflect advocacy proposals. Remote specialists participated through professional collegial arrangements during the study period without direct NHI reimbursement. Sensitivity analyses varying the discount rate (3%-9%), steady-state annual case volume (50-125), and remote specialist fee are reported in Multimedia Appendix 1. Equipment was depreciated on a mixed schedule following the source model: the ultrasound probe and teleconsultation cart over 7 years and all ancillary electronic equipment over 3 years, with corporate income tax (17%) applied to positive pretax profit from the third year onward. A complete depreciation schedule and a year-by-year cash-flow reconciliation for the top-ranked scenario are provided in Tables S5 and S6 of Multimedia Appendix 1. The financial model and the clinical case study data reported here derive from an executive master of business administration (MBA) thesis by HBT [22], a retrospective business-model case study of D Clinic. Reused elements comprise the equipment cost structure, the reimbursement assumptions, and the 24 modeled scenarios (appendix 1 in HBT’s thesis [22]); the examination and teleconsultation counts in Tables 2 and 3 (the table labeled 4-4 in HBT’s thesis [22]); the 4 service domains; and the learning curve observations. This report also adds an independently reproducible year-by-year NPV recomputation with sensitivity analysis and a structured clinical-implementation presentation following the STROBE (Strengthening the Reporting of Observational Studies in Epidemiology) reporting guideline.

### PoCUS Training and Quality Assurance

All 3 clinic physicians (2 in family medicine and 1 in neurology) completed a 40-hour PoCUS training program accredited by the Taiwan Society of Ultrasound in Medicine, consisting of 16 hours of didactic instruction covering cardiac, pulmonary, abdominal, and vascular applications, and 24 hours of supervised hands-on scanning. An additional 8-hour musculoskeletal PoCUS workshop in October 2021, led by a board-certified rehabilitation medicine specialist, expanded the examination repertoire. All 3 physicians performed PoCUS examinations during the study period in approximately equal proportions when on duty. The reported use patterns therefore reflect a multiprovider implementation metric rather than an individual practitioner trajectory. Ongoing quality assurance was maintained through the I-AIM framework, with teleconsultation images reviewed by the remote specialist in real time. No formal competency assessment tool (eg, Objective Structured Assessment of Ultrasound Skills) was applied; image archiving was limited to representative still images shared via the teleconsultation platform.

### Ethical Considerations

This study was conducted in accordance with the Declaration of Helsinki. The PoCUS-teleconsultation service was established in April 2020 as a clinical quality improvement initiative within the home-based medical care program at D Clinic. Throughout the service period (April 2020 to May 2022), examination, teleconsultation, and cost data were recorded as part of routine service documentation and internal quality monitoring rather than under a research protocol. After the service period had ended, the investigators judged that the accumulated implementation experience and financial data had value beyond internal quality improvement, and the activity was accordingly converted into a formal research study. Ethics approval was therefore sought and obtained after data collection had been completed. The study protocol (version 2, July 31, 2023) was reviewed and approved by the National Taiwan University Hospital Research Ethics Committee C through expedited review (202112086RINC) on August 21, 2023, approximately 15 months after the end of the study period. The approval covers the retrospective analysis and publication of the deidentified clinical, operational, and cost data collected between April 2020 and May 2022. Written informed consent for teleconsultation was obtained from all patients or their legal representatives prior to each session, covering both the clinical procedure and the use of anonymized data for quality improvement reporting. Because the service was later converted into a formal research study, the subsequent research ethics committee approval (202112086RINC) specifically covers the retrospective use of these deidentified data for research and publication, extending the original quality improvement consent to the research use reported here. Separate informed consent forms were approved for community and remote medical care, expert consultation meetings, and patient and family participation in teaching recordings. All real-time ultrasound streaming was conducted via encrypted video calls within a password-protected clinical network.

## Results

### PoCUS Use Patterns

**Figure 2:**
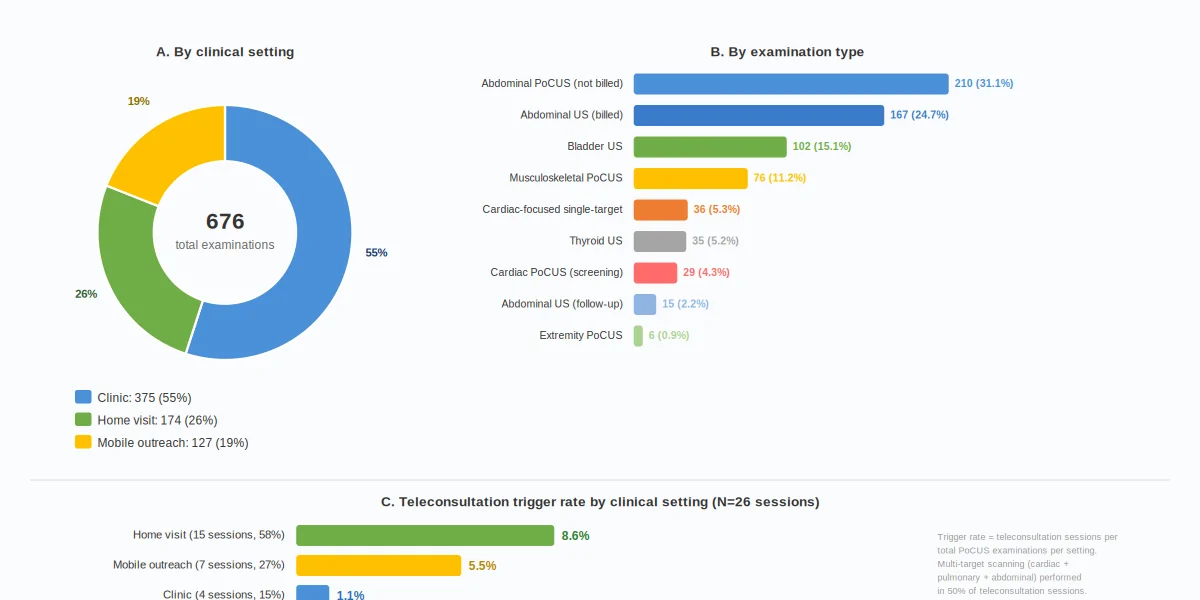
Distribution of point-of-care ultrasound (PoCUS) examinations by clinical setting and examination type (N=676). (A) Donut chart showing distribution across 3 clinical settings; (B) horizontal bar chart of examination types, sorted by frequency; and (C) teleconsultation trigger rate by clinical setting. US: ultrasound.

The 26 patients enrolled in the PoCUS-teleconsultation service had a mean age of 79.4 (SD 9.2) years; 15 (58%) were male. A total of 20 (77%) were in the intensive home health care tier (Barthel Index <35), 4 (15%) were in standard home health care (Barthel Index <60), and 2 (8%) were in palliative home care. The most common primary diagnoses were cerebrovascular disease (n=8, 31%), heart failure (n=6, 23%), and chronic kidney disease (n=4, 15%). During the 26-month study period (April 2020 to May 2022), a total of 676 PoCUS examinations were performed across 3 clinical settings: 375 (55.5%) in clinic, 174 (25.7%) during home visits, and 127 (18.8%) during mobile outreach (Figure 2A). Abdominal examinations constituted the largest category, with 210 (31.1%) unbilled PoCUS screening examinations and 167 (24.7%) billable abdominal ultrasounds (Figure 2B). Bladder ultrasound (n=102, 15.1%), musculoskeletal PoCUS (n=76, 11.2%), thyroid ultrasound (n=35, 5.2%), and cardiac PoCUS (n=29, 4.3% screening and n=36, 5.3% focused, for a total of n=65, 9.6%) accounted for most of the remainder, with follow-up abdominal ultrasound (n=15, 2.2%) and extremity PoCUS (n=6, 0.9%) making up the smallest categories (Table 2). Examinations were logged at the encounter level in the clinic’s routine service record, which did not link individual examinations to patient identifiers; the number of distinct patients contributing to the 676 examinations therefore cannot be established retrospectively. Patient-level characteristics are consequently reported only for the 26 patients who received teleconsultation, for whom individual case records were maintained.

**Table 2:** Point-of-care ultrasound (PoCUS) examinations by clinical setting and examination type (N=676).

Examination type	Clinic (n=375), n (%)	Home visit (n=174), n (%)	Mobile outreach (n=127), n (%)
Abdominal PoCUS (not billed)	101 (26.9)	73 (42)	36 (28.3)
Abdominal ultrasound (billed, 19001C)	100 (26.7)	29 (16.7)	38 (29.9)
Bladder ultrasound	50 (13.3)	42 (24.1)	10 (7.9)
Musculoskeletal PoCUS	54 (14.4)	4 (2.3)	18 (14.2)
Thyroid ultrasound	34 (9.1)	0 (0)	1 (0.8)
Cardiac-focused single-target PoCUS	10 (2.7)	17 (9.8)	9 (7.1)
Cardiac PoCUS (screening)	8 (2.1)	6 (3.4)	15 (11.8)
Abdominal ultrasound (follow-up, 19009C)	12 (3.2)	3 (1.7)	0 (0)
Extremity PoCUS	6 (1.6)	0 (0)	0 (0)

### Teleconsultation Characteristics

Twenty-six teleconsultation sessions were conducted from March 2021 to May 2022: 15 (57.7%) during home visits, 7 (26.9%) during mobile outreach, and 4 (15.4%) in clinic (Table 3). The teleconsultation trigger rate—defined as the proportion of PoCUS examinations prompting specialist consultation—was highest during home visits (15/174, 8.6%), followed by mobile outreach (7/127, 5.5%) and in-clinic examinations (4/375, 1.1%; Figure 2C). Multitarget scanning involving cardiac, pulmonary, and abdominal assessment was performed in 50% (13/26) of teleconsultation sessions. Session duration was typically 30 minutes, with complex dual-specialist consultations extending to 50 minutes. Of the 26 sessions, 24 (92%) were completed without technical interruption. Only 2 (8%) sessions experienced transient 4G connectivity degradation, resulting in temporary image buffering lasting 30 to 90 seconds, but both were completed after signal recovery. No sessions were aborted due to technical failure.

**Table 3:** Teleconsultation session characteristics (N=26).

Characteristics		Sessions, n (%)
**Setting**		
	Home visit	15 (57.7)
	Mobile outreach	7 (26.9)
	Clinic	4 (15.4)
**Specialist type**		
	Cardiologist only	12 (46.2)
	Hospitalist only	6 (23.1)
	Dual specialist (cardiologist+hospitalist)	8 (30.8)
Multitarget scanning (cardiac+pulmonary+abdominal)		13 (50)
**Session duration (minutes)**		
	≤30	18 (69.2)
	31-50	8 (30.8)
**Consultation type**		
	Image interpretation+medication adjustment	18 (69.2)
	Image interpretation only	8 (30.8)
**Teleconsultation trigger rate^a^**		
	Home visit (n=174)	15 (8.6)
	Mobile outreach (n=127)	7 (5.5)
	Clinic (n=375)	4 (1.1)

^a^For the teleconsultation trigger rate rows, percentages are calculated using the total number of point-of-care ultrasound (PoCUS) examinations performed in the respective clinical setting as the denominator because the trigger rate represents the proportion of PoCUS examinations that prompted a teleconsultation.

### Learning Curve Dynamics

Monthly PoCUS volume demonstrated a progressive growth pattern, reaching a sustained plateau of ≥28 examinations per month from August 2021 onward. Three distinct learning phases were observed: (1) an initiation phase (April 2020 to July 2020) characterized by low monthly volume, primarily limited to abdominal and bladder applications; (2) an expansion phase (August 2020 to July 2021) with progressive incorporation of cardiac and thyroid examinations and increasing home-visit use; and (3) a maturation phase (August 2021 to May 2022) marked by sustained high-volume practice, rapid musculoskeletal PoCUS growth following an October 2021 training workshop, and qualitative improvements in diagnostic communication, ultrasound terminology, and the use of proactive provisional diagnoses by on-site physicians during teleconsultation [22].

### Clinical Service Domains

Case-review analysis identified 4 distinct clinical service domains of PoCUS-teleconsultation integration (Figure 1B).

#### Domain 1: HaH Acute Management

PoCUS-guided fluid assessment (cardiac function, pleural effusion, and inferior vena cava dimensions) with remote cardiologist input enabled diuretic titration for acutely decompensated heart failure without hospital transfer. Real-time ultrasound streaming allowed the remote specialist to guide probe positioning and confirm findings before recommending medication adjustments.

#### Domain 2: Home-Based Hospice Multiorgan Support

End-of-life patients with concurrent cardiac and renal failure required dual-specialist teleconsultation (cardiologist and hospitalist). Multitarget PoCUS scanning enabled comprehensive organ assessment to support advance care planning discussions under Taiwan’s Patient Right to Autonomy Act [23]. This domain corresponded to the highest-NPV financial scenario.

#### Domain 3: Green Channel Surgical Referral

PoCUS findings indicating potential surgical emergencies—including septic arthritis with joint effusion, large pericardial effusion, and acute abdominal pathology—triggered expedited “green channel” transfer to Taitung Mackay Memorial Hospital. PoCUS-documented findings accompanying the referral facilitated prearrival surgical planning and reduced emergency department triage time.

#### Domain 4: Chronic Disease Serial Monitoring

Serial PoCUS examinations (cardiac function, pleural effusion, inferior vena cava dimensions, and bladder residual volume) provided objective longitudinal data for chronic disease medication titration, complementing the NHI pay-for-performance programs for diabetes, chronic kidney disease, chronic obstructive pulmonary disease, and hepatitis B or C.

### Financial Analysis

No adverse events attributable to the PoCUS-teleconsultation workflow were identified during the study period. No cases of delayed diagnosis, missed surgical emergency, or medication error resulting from teleconsultation-guided adjustment were documented. However, formal adverse event tracking was not implemented as a structured protocol.

**Figure 3:**
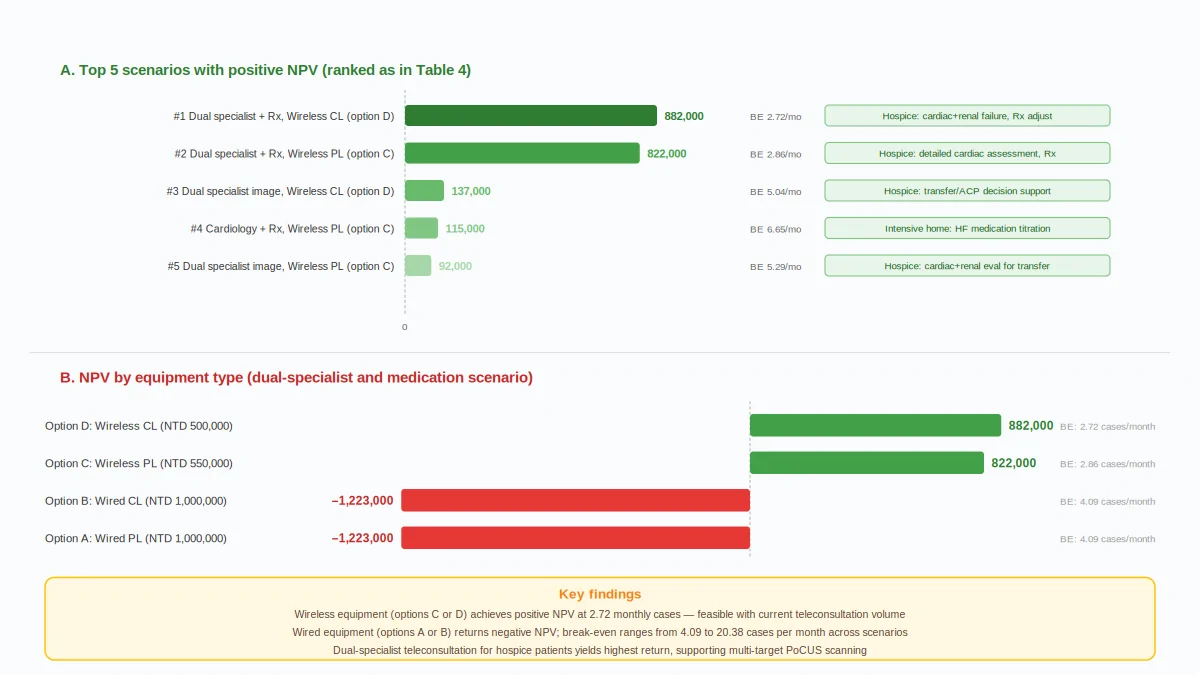
Seven-year net present value (NPV) analysis of point-of-care ultrasound (PoCUS)–teleconsultation scenarios. (A) Top 5 scenarios with positive NPV, ranked as reported in Table 4, with corresponding clinical applications. (B) NPV comparison across 4 equipment options under the optimal dual-specialist and medication adjustment (Rx) scenario. The discount rate was 6%. Green bars indicate positive NPV, and red bars indicate negative NPV. BE: break-even; CL: convex+linear; HF: heart failure; PL: phased-array+linear.

NPV analysis across 24 scenarios revealed a clear bifurcation between wireless and wired equipment viability (Figure 3). The top 5 positive-NPV scenarios all involved wireless PoCUS equipment (Table 4); the complete set of 24 scenarios is reported in Multimedia Appendix 2. The highest-NPV scenario—dual-specialist teleconsultation with medication adjustment using wireless convex and linear probes (option D)—yielded an NT $882,000 NPV over 7 years with a break-even threshold of only 2.72 monthly cases, a volume consistent with the observed teleconsultation pattern. Single-specialist cardiology teleconsultation with medication adjustment using wireless phased-array and linear probes (option C) achieved a positive NPV with a break-even threshold of 6.65 monthly cases—achievable with moderate use growth. In contrast, every wired equipment scenario (options A and B) returned a negative NPV, with break-even thresholds ranging from 4.09 to 20.38 monthly cases, rendering them financially unfavorable under the modeled assumptions at current or projected use levels.

**Table 4:** Top 5 positive net present value (NPV) scenarios for point-of-care ultrasound—teleconsultation integration^a^.

Rank	Consultation type	Equipment	NPV (NT $^b^)	Break-even volume (cases/month)	Primary clinical application
1	Dual specialist+Rx^c^	Option D: wireless CL^d^	882,000	2.72	Hospice: cardiac and renal failure and medication adjustment
2	Dual specialist+Rx	Option C: wireless PL^e^	822,000	2.86	Hospice: detailed cardiac assessment and medication adjustment
3	Dual-specialist image	Option D: wireless CL	137,000	5.04	Hospice: transfer decision or advance care planning
4	Cardiology+Rx	Option C: wireless PL	115,000	6.65	Intensive home care: heart failure medication titration
5	Dual-specialist image	Option C: wireless PL	92,000	5.29	Hospice: cardiac and renal evaluation for transfer decisions

^a^Discount rate was 6%. Depreciation followed a mixed schedule, with the probe and cart depreciated over 7 years and ancillary equipment over 3 years (Table S5 in Multimedia Appendix 1). All NPVs and break-even figures were independently recomputed from the source cost structure and reproduced the source model, except for the rank 4 break-even value (6.65 monthly cases), which was taken directly from the source text and was not independently recomputed because the underlying variable-cost rows were incompletely legible in the source document.

^b^NT $1=US $0.036 in 2021 on average.

^c^Rx: medication adjustment.

^d^CL: convex+linear.

^e^PL: phased-array+linear.

Under the existing NHI reimbursement structure—in which multitarget bedside examinations fall only under a generic “other ultrasound” code (19005C, 600 NHI points), with no dedicated multitarget or B2B specialist fees—equipment cost recovery would require approximately 14.6 years, exceeding the equipment depreciation horizon and underscoring the urgent need for reimbursement reform.

## Discussion

### Principal Findings

This study represents, to our knowledge, the first report of integrated PoCUS-teleconsultation implementation within a dedicated home-based medical care practice in an Asian setting and the first to provide financial modeling for such integration under different reimbursement scenarios. Our findings extend the existing evidence base in several important dimensions.

### Comparison With Existing Evidence

Our 676-examination dataset represents the largest reported PoCUS use series in an Asian home-based care context. The observed teleconsultation trigger rate of 8.6% during home visits aligns with the concept of PoCUS as a diagnostic triage tool—identifying cases requiring specialist input while enabling most examinations to inform on-site clinical decisions independently. This pattern is consistent with findings by Kirkpatrick et al [15] that novice operators can achieve diagnostic-quality PoCUS images in home settings with remote guidance and supports the broader tele-ultrasound evidence synthesized by Recker et al [24].

The progressive learning curve that reached a sustained plateau of 28 or more monthly examinations by month 17 of implementation is notable. Although formal competency frameworks, such as I-AIM, define individual skill acquisition milestones [12,13], our data suggest that practice-level adoption follows a distinct trajectory characterized by volume growth, scope expansion (particularly musculoskeletal applications postworkshop training), and qualitative communication maturation during teleconsultation encounters.

International comparison supports the relevance of these findings. A Spanish HaH PoCUS study [25] reported 85 ultrasounds among 72 patients, with hospital displacement avoided in 73% of cases, and the Canadian ACCUMEN-POCUS (Aiding Chronic Obstructive Pulmonary Disease and Congestive Heart Failure Ultrasound-Guided Management Through Enhanced Point-of-Care Ultrasound) trial [26] represents the first randomized controlled trial protocol evaluating remotely interpreted PoCUS in HaH settings. A 2024 scoping review of tele-ultrasound [27] identified connectivity, training, and regulatory frameworks as key enablers, consistent with the barriers observed in our implementation.

### The B2B2C Model: Policy Implications

Our B2B2C teleconsultation model addresses a structural gap in existing telemedicine frameworks, which predominantly assume a direct physician-to-patient (B2C) or physician-to-physician referral (B2B) paradigm. The integrated model—where an on-site generalist performs PoCUS, streams findings to a remote specialist who provides diagnostic and therapeutic guidance that is communicated back to the patient—creates value at multiple levels consistent with the Porter high-value health care framework [28]. For patients, the model avoids the physical, emotional, and financial burden of hospital transfer; for the health care system, it may reduce emergency department use and inpatient admissions; and for clinicians, it enables knowledge transfer and progressive skill development.

South Korea’s experience with dedicated PoCUS reimbursement codes provides evidence supporting the role of reimbursement policy in driving use [20,21]. Kang et al [20] demonstrated a 4-fold increase in emergency department PoCUS use following the introduction of STU and MTU codes in July 2019. Taiwan’s NHI system should consider adopting analogous codes to incentivize PoCUS integration in home-based care settings.

The 2024 amendments removing geographic restrictions on telemedicine were enacted after the study period (2020 to 2022). Our operational data were generated under the pre-2024 regulatory framework, which restricted teleconsultation to patients in designated remote areas, long-term care facilities, or emergencies. The financial model applicability may differ under the expanded 2024 framework, where eligibility is broader, but reimbursement structures may also evolve.

### Challenges and Barriers

Several challenges were identified during implementation. First, the absence of dedicated NHI reimbursement for multitarget bedside ultrasound and B2B specialist teleconsultation represents the primary barrier to financial sustainability. The current generic code (19005C, 600 points) grossly undervalues the clinical and time investment required for comprehensive cardiac, pulmonary, and abdominal PoCUS assessment with real-time specialist guidance. Second, 4G network latency in mountainous Taitung terrain occasionally compromised real-time image streaming quality; Taiwan’s 5G infrastructure expansion pilot in Taitung offers a potential solution [29]. Third, the limited pool of specialists willing to participate in B2B teleconsultation—particularly during nonbusiness hours—constrains service scalability. Finally, I-AIM documentation requirements add approximately 5 to 10 minutes per examination, which may limit adoption in time-pressured home-visit contexts.

The study period (April 2020 to May 2022) overlapped substantially with the COVID-19 pandemic, which may have influenced findings in several ways. Temporary NHI regulatory relaxations expanded telemedicine eligibility, potentially increasing clinician willingness to adopt teleconsultation. Infection control concerns may have increased preference for remote specialist input over in-person referrals. Conversely, pandemic-related travel restrictions reduced mobile outreach capacity during local outbreak periods. The extent to which the normalization of postpandemic regulations will affect teleconsultation demand remains uncertain.

### Strengths and Limitations

This study has several strengths: real-world implementation in a resource-constrained rural setting, a substantial PoCUS use dataset (676 examinations over 26 months), and an integrated financial analysis framework. However, several limitations should be noted. First, the single-site design with 3 operators limits external validity, although the multiprovider implementation strengthens the generalizability of the learning curve observations compared with those of a single-operator study. Second, the small teleconsultation sample (n=26, 3.8% of total examinations) constrains conclusions about teleconsultation scalability and effectiveness. Third, D Clinic is a dedicated home-based medical care practice; traditional rural clinics would face substantially different operational barriers to implementing this model. Fourth, examinations were recorded at the encounter level without linkage to patient identifiers, so the number of unique patients underlying the 676 examinations could not be determined; use is therefore expressed as examinations rather than as examinations per patient, and the extent of repeat scanning in individual patients is unknown. Fifth, the financial analysis relies on proposed reimbursement rates not yet adopted by NHI, making the projections hypothetical. Sixth, adverse event tracking was observational rather than protocol-driven. Seventh, confirmatory diagnosis rates for PoCUS-triggered surgical referrals were not tracked. Eighth, the study period overlapped with the COVID-19 pandemic, which may have influenced teleconsultation adoption. Finally, patient-reported outcome measures (PROMs) and patient-reported experience measures (PREMs) were not collected.

### Future Directions

Although these findings suggest potential policy directions, including dedicated NHI reimbursement codes for multitarget bedside ultrasound and B2B teleconsultation fees, the absence of controlled clinical outcome data means that these recommendations should be considered preliminary. Rigorous multisite studies with patient-level outcome measurement, formal cost-effectiveness analysis, and comparison with standard care pathways are needed before advocating for national reimbursement policy changes. Future work should also evaluate the model across diverse rural settings with varying digital infrastructure, physician density, and patient demographics.

### Conclusions

This pilot study demonstrates the feasibility of integrating PoCUS into rural home-based medical care, with 676 examinations across 3 clinical settings over 26 months. The teleconsultation component, while limited to 26 sessions (3.8% of total examinations), illustrated the operational workflow and potential clinical utility of the B2B2C model. Financial scenario modeling suggests favorable projected returns for wireless equipment configurations under proposed but not yet implemented reimbursement structures. These findings should be interpreted as hypothesis generating and as a basis for future multisite studies with patient-level outcome measurement rather than as definitive evidence of clinical effectiveness or financial viability.

## Data Availability

Anonymized aggregate data supporting this study are available from the corresponding author upon reasonable request. Individual patient data cannot be shared due to privacy regulations under Taiwan’s Personal Data Protection Act.

## Appendix

Multimedia Appendix 1 Supplementary tables, including the net present value of all 24 equipment reimbursement scenarios (Table S1); sensitivity analysis of net present value across varying discount rates, case volumes, and remote specialist fee (Table S2); the evolution of Taiwan’s telemedicine regulatory framework (Table S3); the STROBE (Strengthening the Reporting of Observational Studies in Epidemiology) checklist (Table S4); and detailed model assumptions.

Multimedia Appendix 2 Seven-year net present value analysis of all 24 modeled scenarios, including the source citation, model assumptions, and the record of verification against the source financial model (Microsoft Excel workbook).

## Abbreviations

ACCUMEN-POCUS: Aiding Chronic Obstructive Pulmonary Disease and Congestive Heart Failure Ultrasound-Guided Management Through Enhanced Point-of-Care Ultrasound
B2B: business-to-business
B2B2C: business-to-business-to-consumer
B2C: business-to-consumer
HaH: hospital-at-home
I-AIM: indication, acquisition, interpretation, and medical decision-making
MBA: master of business administration
MTU: multitarget ultrasound
NHI: National Health Insurance
NPV: net present value
PoCUS: point-of-care ultrasound
PREM: patient-reported experience measure
PROM: patient-reported outcome measure
STROBE: Strengthening the Reporting of Observational Studies in Epidemiology
STU: single-target ultrasound
