# Supplementary material for: Point-of-Care Ultrasound Integrated With Teleconsultation for Rural Home-Based Medical Care: Pilot Implementation and Financial Analysis
Source: JMIR Med Inform. 2026 Aug 28;14:e93876. doi: 10.2196/93876 (PMC13570238; doi:10.2196/93876)
Supplement: Supplementary file 1 [file medinform_v14i1e93876_app1.docx]

**Multimedia Appendix 1**

Supplementary Tables and Methods

**Table S1.** Net Present Value of All 24 Equipment–Reimbursement Scenarios Over the 7-Year Analysis Horizon (NTD Thousands)

| **Consultation Type** | **Option A Wired PL** | **Option B Wired CL** | **Option C Wireless PL** | **Option D Wireless CL** |
| --- | --- | --- | --- | --- |
| Cardiology + Rx (image+medication) | -504 (9.50) | -504 (9.50) | +115 (6.65) | -41 (6.33) |
| Hospitalist + Rx (image+medication) | -473 (9.01) | -473 (9.01) | -48 (6.30) | -3 (6.00) |
| Dual specialist + Rx (image+medication) | -1223 (4.09) | -1223 (4.09) | +822 (2.86) | +882 (2.72) |
| Cardiology image only | -951 (20.38) | -750 (16.09) | -57 (14.25) | -12 (13.57) |
| Hospitalist image only | -907 (18.25) | -907 (18.25) | -457 (12.76) | -407 (12.15) |
| Dual specialist image only | -324 (7.57) | -160 (6.31) | +92 (5.29) | +137 (5.04) |

*Values shown as NPV in NTD thousands (break-even monthly cases), taken from the source financial model [22] and reproduced in full, with the verification record, in Multimedia Appendix 2. Rx indicates medication adjustment; PL, phased+linear; CL, convex+linear. Discount rate 6%. Equipment is depreciated on a mixed schedule: the ultrasound probe and teleconsultation cart over 7 years, and the tablet, laptop, mobile network card, video camera, and charging station over 3 years (see Supplementary Methods and Table S5). Options A and B carry the same equipment cost (NTD 1,000,000); the source sheets nevertheless report different values for the two image-only consultation types, and the underlying cost rows for those four scenarios differ accordingly.*

**Table S2. Sensitivity Analysis of Net Present Value Under Varying Assumptions (Top-Ranked Scenario: Dual Specialist + Rx, Wireless CL, Option D)**

| **Parameter** | **Value** | **NPV (NTD K)** | **Break-Even (cases/mo)** |
| --- | --- | --- | --- |
| Discount Rate |  |  |  |
|  | 3% | +1,088 | 2.72 |
|  | 5% | +947 | 2.72 |
|  | 6% (baseline) | +882 | 2.72 |
|  | 7% | +821 | 2.72 |
|  | 9% | +709 | 2.72 |
| Steady-State Annual Case Volume |  |  |  |
|  | 50 cases/yr | +357 | 2.72 |
|  | 75 cases/yr | +619 | 2.72 |
|  | 100 cases/yr (baseline) | +882 | 2.72 |
|  | 125 cases/yr | +1,145 | 2.72 |
| Remote Specialist Payout |  |  |  |
|  | Baseline payout (NTD 1146 per session) | +882 | 2.72 |
|  | +50% (NTD 1719 per session) | +669 | 3.14 |

All values were recomputed from the source financial model [22], whose baseline this model reproduces exactly (NPV NTD +882,155 at 6%; break-even 2.72 cases per month). Assumptions: equipment cost NTD 500,000 for the wireless convex+linear probe (Option D), depreciated over 7 years, with ancillary equipment depreciated over 3 years (full schedule in Table S5); revenue NTD 7200 per session; variable cost NTD 2848 per session; annual fixed cost NTD 142,292; corporate tax 17% applied from year 3 onward, following the source model. Case volumes for years 1 to 3 are held at the observed and projected values of 26, 57 and 80; only the steady-state volume from year 4 is varied. Break-even is unaffected by the discount rate or by case volume because it depends only on fixed cost and contribution margin. The remote specialist payout (NTD 1146 per session) is a modelled cost representing compensation to remote specialists under the proposed reimbursement structure; it is distinct from the remote specialist teleconsultation fee treated as revenue in the Supplementary Methods. No such payout was made during the pilot itself, in which remote specialists participated through professional collegial arrangements without direct National Health Insurance reimbursement; the payout applies only to the modelled future scenario.

**Table S3.** Evolution of Taiwan's Telemedicine Regulatory Framework (1986–2024)

| **Year** | **Regulatory Action** | **Key Provisions / Impact** |
| --- | --- | --- |
| **1986** | Physicians Act Article 11 (original) | Established principle of in-person diagnosis requirement; no telemedicine provision |
| **2001** | DOH Pilot Telemedicine Projects | Demonstration projects in offshore islands (Penghu, Kinmen, Matsu) and mountain townships |
| **2018** | MOHW Telemedicine Regulations | Permitted teleconsultation for remote areas, long-term care facilities, emergency situations, and chronic disease follow-up within 3 months |
| **2019** | NHI Teleconsultation Codes | Created reimbursement codes for physician-to-patient teleconsultation; 2330 claims filed nationally |
| **2020** | COVID-19 Emergency Measures | Temporary expansion to quarantined individuals; regulatory flexibility for pandemic response |
| **2021** | Delta Wave Expansion | NHI teleconsultation claims surged to approximately 35,000 |
| **2022** | Omicron Wave Peak | NHI claims reached approximately 169,044 (72-fold increase from 2019); permanent regulatory relaxation proposed |
| **2024** | Comprehensive Revision | Geographic restrictions removed; teleconsultation permitted for initial visits in any setting |

# Supplementary Methods: Detailed NPV Model Assumptions

*Revenue streams:* Each teleconsultation session generated revenue from 2 components: (1) on-site multi-target PoCUS examination fee (proposed NTD 1200) and (2) remote specialist teleconsultation fee (NTD 3000 for image interpretation and medication adjustment, or NTD 2000 for image interpretation only). Dual-specialist sessions generated 2 specialist fees.

*Cost structure:* Equipment acquisition costs: Option A (wired phased+linear), NTD 1,000,000; Option B (wired convex+linear), NTD 1,000,000; Option C (wireless phased+linear), NTD 550,000; Option D (wireless convex+linear), NTD 500,000. Equipment was depreciated using the straight-line method on a mixed schedule: the ultrasound probe and teleconsultation cart over 7 years, and all ancillary electronic equipment over 3 years (full schedule in Table S5). Annual fixed costs comprise probe depreciation, the teleconsultation cart, tablet, laptop, mobile network card, video camera and charging station, together with platform maintenance of NTD 36,000 annually, totalling NTD 142,292 for Option D and NTD 149,435 for Option C. Variable costs of NTD 2848 per session cover the on-site physician, the remote specialist or specialists, case management, gel, disinfectants, sterile sheaths and consumables.

*Case volume projections:* Year 1 volume (26 cases) based on actual observed sessions. Year 2 (57 cases) assumed continued linear growth. Year 3 (80 cases) assumed approach to steady-state. Years 4 to 7 (100 cases/year) assumed steady-state operations. Discount rate: 6%, as used in the source financial model [22].

**Table S4. STROBE Checklist**

STROBE Statement—Checklist of Items That Should Be Included in Reports of Observational Studies

| Item | No | Recommendation | Reported on Page / Section |
| --- | --- | --- | --- |
| Title and abstract | 1 | (a) Indicate the study's design with a commonly used term in the title or the abstract | Title, Abstract |
|  |  | (b) Provide in the abstract an informative and balanced summary of what was done and what was found | Abstract |
| Background/rationale | 2 | Explain the scientific background and rationale for the investigation being reported | Introduction, paragraphs 1-6 |
| Objectives | 3 | State specific objectives, including any prespecified hypotheses | Introduction, final paragraph |
| Study design | 4 | Present key elements of study design early in the paper | Methods: Study Design and Setting |
| Setting | 5 | Describe the setting, locations, and relevant dates, including periods of recruitment, exposure, follow-up, and data collection | Methods: Study Design and Setting |
| Participants | 6 | Give the eligibility criteria, and the sources and methods of selection of participants | Methods: Patient Population and Care Tiers |
| Variables | 7 | Clearly define all outcomes, exposures, predictors, potential confounders, and effect modifiers | Methods: Data Collection and Outcomes |
| Data sources/measurement | 8 | For each variable of interest, give sources of data and details of methods of assessment (measurement) | Methods: Data Collection and Outcomes; Financial Analysis |
| Bias | 9 | Describe any efforts to address potential sources of bias | Discussion: Strengths and Limitations |
| Study size | 10 | Explain how the study size was arrived at | Methods: Study Design (clinic patient panel ~90) |
| Quantitative variables | 11 | Explain how quantitative variables were handled in the analyses | Methods: Financial Analysis |
| Statistical methods | 12 | (a) Describe all statistical methods, including those used to control for confounding | Methods: Financial Analysis (NPV methodology) |
|  |  | (b) Describe any methods used to examine subgroups and interactions | N/A (descriptive study) |
|  |  | (c) Explain how missing data were addressed | N/A (complete data for all PoCUS records) |
|  |  | (d) If applicable, describe analytical methods taking account of sampling strategy | N/A |
|  |  | (e) Describe any sensitivity analyses | Multimedia Appendix 1 (discount rate sensitivity) |
| Participants | 13 | (a) Report numbers of individuals at each stage of study | Results: PoCUS Utilization Patterns. The number of distinct patients contributing to the 676 examinations cannot be established retrospectively because logging was at the encounter level (see Results and Limitations); patient-level data are reported for the 26 teleconsultation sessions. |
|  |  | (b) Give reasons for non-participation at each stage | N/A (all examinations included) |
|  |  | (c) Consider use of a flow diagram | N/A |
| Descriptive data | 14 | (a) Give characteristics of study participants and information on exposures and potential confounders | Results; Tables 2-3. Participant characteristics are available for the 26 teleconsultation patients; the number of distinct patients underlying the 676 examinations cannot be established retrospectively. |
|  |  | (b) Indicate number of participants with missing data for each variable of interest | N/A (complete data) |
| Outcome data | 15 | Report numbers of outcome events or summary measures | Results: all subsections; Tables 2-4 |
| Main results | 16 | (a) Give unadjusted estimates and, if applicable, confounder-adjusted estimates | Results: Financial Analysis; Table 4 |
|  |  | (b) Report category boundaries when continuous variables were categorized | Methods: Care Tiers (Barthel Index <60) |
|  |  | (c) If relevant, consider translating estimates into meaningful clinical time period | Results: break-even monthly cases |
| Other analyses | 17 | Report other analyses done—eg analyses of subgroups and interactions, and sensitivity analyses | Results: 24-scenario NPV; Multimedia Appendix 1 |
| Key results | 18 | Summarize key results with reference to study objectives | Discussion: Principal Findings |
| Limitations | 19 | Discuss limitations of the study, taking into account sources of potential bias or imprecision | Discussion: Strengths and Limitations |
| Interpretation | 20 | Give a cautious overall interpretation of results considering objectives, limitations, multiplicity of analyses, results from similar studies, and other relevant evidence | Discussion: Comparison With Existing Evidence; B2B2C Model |
| Generalizability | 21 | Discuss the generalizability (external validity) of the study results | Discussion: Strengths and Limitations (single-center) |
| Funding | 22 | Give the source of funding and the role of the funders for the present study | Funding section |

Adapted for: "Point-of-Care Ultrasound Integrated With Teleconsultation for Rural Home-Based Medical Care"

STROBE indicates Strengthening the Reporting of Observational Studies in Epidemiology. This checklist is adapted from von Elm E, Altman DG, Egger M, et al. The Strengthening the Reporting of Observational Studies in Epidemiology (STROBE) Statement: guidelines for reporting observational studies. Ann Intern Med. 2007;147(8):573-577. PMID:17938396

**Table S5. Equipment Depreciation Schedule for the Top-Ranked Scenario (Dual Specialist + Rx, Option D)**

| **Equipment Item** | **Acquisition Cost (NTD)** | **Depreciation Period** | **Annual Depreciation (NTD)** |
| --- | --- | --- | --- |
| Ultrasound probe (wireless convex+linear) | 500,000 | 7 years | 71,429 |
| Teleconsultation cart | 42,000 | 7 years | 6,000 |
| Android 5G tablet | 21,900 | 3 years | 7,300 |
| Windows laptop | 42,600 | 3 years | 14,200 |
| 5G mobile network card | 9,900 | 3 years | 3,300 |
| Video camera | 3,200 | 3 years | 1,067 |
| Charging station | 8,990 | 3 years | 2,997 |
| Total equipment investment | 628,590 | — | 106,292 |

*The ultrasound probe and teleconsultation cart are depreciated over 7 years; all ancillary electronic equipment is depreciated over 3 years. Annual platform maintenance of NTD 36,000 is expensed rather than depreciated. Total annual fixed cost is therefore NTD 106,292 (depreciation) + NTD 36,000 (platform maintenance) = NTD 142,292. Individual annual depreciation values are rounded to the nearest NTD and sum to NTD 106,293; the total shown (NTD 106,292) is computed from the unrounded values (NTD 106,291.91) and is the figure used in the model.*

**Table S6. Year-by-Year Cash-Flow Analysis for the Top-Ranked Scenario (Dual Specialist + Rx, Option D; NTD)**

| **Year** | **Cases** | **Revenue** | **Fixed Cost** | **Variable Cost** | **Depreciation** | **Pretax Profit** | **Tax (17%)** | **Net Profit** | **Cash Flow** | **Discounted CF** |
| --- | --- | --- | --- | --- | --- | --- | --- | --- | --- | --- |
| 1 | 26 | 187,200 | 142,292 | 74,051 | 106,292 | −29,143 | 0 | −29,143 | 77,149 | 72,782 |
| 2 | 57 | 410,400 | 142,292 | 162,342 | 106,292 | 105,766 | 0 | 105,766 | 212,058 | 188,731 |
| 3 | 80 | 576,000 | 142,292 | 227,848 | 106,292 | 205,860 | 34,996 | 170,864 | 277,156 | 232,705 |
| 4 | 100 | 720,000 | 142,292 | 284,810 | 106,292 | 292,898 | 49,793 | 243,105 | 349,397 | 276,755 |
| 5 | 100 | 720,000 | 142,292 | 284,810 | 106,292 | 292,898 | 49,793 | 243,105 | 349,397 | 261,090 |
| 6 | 100 | 720,000 | 142,292 | 284,810 | 106,292 | 292,898 | 49,793 | 243,105 | 349,397 | 246,311 |
| 7 | 100 | 720,000 | 142,292 | 284,810 | 106,292 | 292,898 | 49,793 | 243,105 | 349,397 | 232,369 |

*Revenue = NTD 7200 per session (NTD 1200 on-site examination + NTD 6000 dual-specialist remote fee). Variable cost = NTD 2848 per session. Fixed cost includes depreciation (Table S5) plus platform maintenance. Following the source model, corporate income tax (17%) is applied to positive pretax profit from year 3 onward; years 1 and 2 are untaxed. Cash flow = net profit + depreciation add-back. Discounted at 6%. Net present value = −628,590 (initial investment) + Σ discounted cash flows = NTD +882,155, reproducing the headline figure exactly.*

# References

*References for this Multimedia Appendix correspond to those in the main manuscript.*
